# Microbiota and Methylglyoxal-Derived AGEs: Implications in Ageing and Age-Related Disease

**DOI:** 10.3390/life16081265

**Published:** 2026-07-30

**Authors:** Niki Tombolesi, Emanuele Francini, Gretta Veronica Badillo-Pazmay, Carlo Fortunato, Francesca Marchegiani, Fabiola Olivieri, Stefania Fumarola, Rosanna Maniscalco, Giulia Matacchione

**Affiliations:** 1 Department of Biomedical Sciences and Public Health, Marche Polytechnic University, Marche University Hospital, 60121 Ancona, Italy; niki.tombolesi@ospedaliriuniti.marche.it; 2 Clinic of Laboratory and Precision Medicine, IRCCS INRCA, 60121 Ancona, Italy; e.francini@inrca.it (E.F.); g.matacchione@inrca.it (G.M.); 3 Center of Biogerontology, IRCCS INRCA, 60121 Ancona, Italyf.olivieri@univpm.it (F.O.); s.fumarola@inrca.it (S.F.); 4 Laboratory of Experimental Pathology, Department of Clinical and Molecular Sciences, Università Politecnica delle Marche, 60100 Ancona, Italy; 5 Department of Biomolecular Sciences, University of Urbino Carlo Bo, 61029 Urbino, Italy; r.maniscalco@inrca.it

**Keywords:** methylglyoxal, advanced glycation end products, gut microbiota, dicarbonyl stress, ageing, inflammaging, intestinal permeability, dysbiosis, glyoxalase system, age-related diseases

## Abstract

Ageing is characterized by progressive metabolic and inflammatory dysregulation, in which methylglyoxal (MGO), a highly reactive dicarbonyl by-product of glycolysis, is involved in the formation of advanced glycation end products (AGEs). This review provides an integrated overview of the bidirectional relationship between MGO-derived carbonyl stress and the gut microbiota, focusing on its implications for ageing and age-related diseases. We summarize current evidence on MGO production, clearance, tissue distribution, and reactivity, with particular attention to the intestinal lumen as a site where dietary compounds, host metabolism, and microbial activity converge. Age-related dysbiosis may impair intestinal barrier integrity, alter microbial metabolite production, and promote chronic low-grade inflammation, thereby reinforcing metabolic dysfunction and favoring free MGO accumulation. Conversely, MGO and AGEs can reshape microbial communities, compromise epithelial tight junctions, and amplify inflammatory signalling through receptor-dependent and independent mechanisms. Evidence from in vitro, animal, and clinical studies supports a role for the MGO–microbiota axis in metabolic, cardiovascular, gastrointestinal, neurodegenerative, and frailty-related conditions. Targeting microbiota composition, intestinal barrier function, and MGO scavenging pathways may therefore represent a promising strategy to mitigate carbonyl stress and preserve health during ageing.

## 1. Introduction

Methylglyoxal (MGO) is a highly cytotoxic α-ketoaldehyde endogenously generated as a byproduct of glycolysis through the degradation of the triose phosphates glyceraldehyde-3-phosphate (GAP) and dihydroxyacetone phosphate (DHAP). MGO is recognized as a central mediator of dicarbonyl stress, an unbalanced condition characterized by the accumulation of highly reactive carbonyl intermediates. It readily and irreversibly modifies proteins, lipids, and nucleic acids, leading to the formation of advanced glycation end-products (AGEs) [1]. Under homeostatic conditions, MGO clearance is primarily ensured by the glutathione-dependent glyoxalase system, which converts MGO into harmless D-lactate through two consecutive enzymatic steps [2]. However, the effectiveness of this protective mechanism decreases during the aging process and in pathological states, leading to dicarbonyl dysregulation and an increasing concentration of free MGO in intracellular and extracellular spaces. This disruption negatively affects tissue architecture and activity by activating various signaling cascades via both receptor-linked and non-receptor-linked mechanisms. Consequently, MGO and its derived AGEs contribute to chronic low-grade inflammation, endothelial dysfunction, and progressive tissue injury, thereby accelerating the functional decline associated with ageing [3,4].

In recent years, increasing evidence has highlighted a strong bidirectional interaction between MGO and the gut microbiota (GM). The GM constitutes a dynamic and complex community of microorganisms in constant crosstalk with the host, regulating metabolic, immune, and inflammatory pathways [5]. As people age, the gut microbiota experiences dysbiosis, which is characterized by shifts in its makeup and operation, including decreased microbial variety and diminished bioactive metabolite synthesis. This reorganization of the gut’s microbial community is linked to greater intestinal permeability, the persistent low-level inflammation termed inflammaging, and metabolic imbalances [6], all of which are established indicators of the aging process. The role of the gut microbiota in ageing, or the involvement of MGO and AGEs in metabolic diseases is well-known, but a comprehensive synthesis explicitly linking dicarbonyl stress to the gut microbiota within the context of senescence is currently lacking. In this perspective, the GM emerges as a central hub in the metabolism–inflammation–aging axis, contributing directly or indirectly to the regulation of MGO levels. The concept of an MGO-GM axis is therefore gaining increasing recognition, in which dysbiosis and host metabolic dysregulation establish a self-perpetuating vicious cycle that sustains carbonyl stress and systemic inflammation. Within this network, both endogenous free MGO accumulation and dietary AGEs exposure reshape the bacterial taxonomy, leading to a depletion of beneficial taxa and a relative enrichment of less favorable microbial populations. These alterations compromise epithelial barrier integrity, disrupt incretin hormone secretion, and, through perturbation of the bile acid pool, contribute to the progression of metabolic and vascular disorders, including insulin resistance, metabolic dysfunction-associated steatotic liver disease (MASLD), and vascular damage [7]. The gastrointestinal tract thus plays a dual role, acting simultaneously as a source and a target of glycation stress. In addition, microbial metabolism itself may contribute to local MGO generation, further affecting luminal homeostasis and intestinal permeability. Finally, in the context of ageing, this axis actively contributes to multiorgan functional decline. In the brain, AGE accumulation in microglial cells promotes mitochondrial dysfunction and neuroinflammation associated with cognitive impairment, whereas in skeletal muscle MGO disrupts energy metabolism by interfering with the Krebs cycle and inhibiting myogenic differentiation through downregulation of key transcriptional regulators [8].

The objective of this review is to provide a critical and integrated overview of the MGO–microbiota axis, exploring its key role in metabolic diseases and ageing. By combining the study of the molecular mechanisms of dicarbonyl stress with the analysis of intestinal barrier integrity and its systemic consequences, this review moves beyond the conception of MGO-induced stress and dysbiosis as independent clinical entities. This review provides a novel conceptual framework that focuses on the MGO-GM crosstalk as a cooperative, bidirectional pathogenetic circuit, crucial for the development of therapeutic strategies and nutritional interventions aimed at mitigating age-related frailty.

## 2. Methods

This narrative review was written in accordance with the Scale for the Quality Assessment of Narrative Review Articles (SANRA) guidelines [9]. The bibliographic research was performed on PubMed and Web of Science databases up to June 2026 using “MGO”, “dicarbonyl stress”, “gut microbiota”, “dysbiosis”, “aging”, and “inflammaging” as the primary search queries.

## 3. Production, Reactivity, Clearance and Tissue Distribution of Methylglyoxal

MGO is one of the major reactive dicarbonyl intermediates generated during cellular metabolism [7]. From a chemical perspective, MGO is defined by two highly reactive electrophilic carbonyl groups that easily bind with nucleophilic targets like amino and thiol groups in proteins [10], as well as with lipids and nucleic acids [11]. Because it reacts significantly faster than glucose, MGO is a central driver of intracellular glycation and is considered a key precursor to AGEs [12], which are major contributors to dicarbonyl stress. The primary source of MGO in humans is the spontaneous degradation of the glycolytic trioses phosphates, GAP and DHAP. These intermediates can undergo spontaneous phosphate elimination, resulting in MGO formation [13]. Under physiological conditions, glycolytic flux is poorly diverted towards MGO production.

However, conditions like hyperglycaemia, elevated glycolytic flux, or downstream pathway blockages (e.g., via the inhibition of the key glycolytic enzyme GAPDH) trigger the accumulation of triose phosphates.

This event leads to a proportional increase in the rate of MGO production. Therefore, the latter can be considered as a sensitive indicator of metabolic imbalance: any condition that increases glycolytic flux or alters redox balance (e.g., elevated NADH levels or oxidative stress) can indirectly promote its formation.

Additional metabolic pathways may also contribute to MGO production, including aminoacetone metabolism, lipid peroxidation, and ketone body metabolism [11]. Although quantitatively less relevant than glycolytic sources, these paths assume greater relevance in pathological states characterized by redox alterations and chronic inflammation.

In addition to the non-enzymatic pathway, an enzymatic via for MGO formation mediated by methylglyoxal synthase (MGS) has been described. This enzyme, extensively characterized in bacteria such as *Escherichia coli*, catalyses the conversion of DHAP into MGO and subsequently into D-lactate under conditions of glycolytic stall or reduced inorganic phosphate availability. In this way, the pathway temporarily by-passes metabolic block and ensure cell survival [14].

The significance of analogous enzymatic processes in humans remains poorly understood. Despite extensive research, a human gene for a protein homologous to bacterial MGS has yet to be found. However, alternative enzymatic pathways that may generate MGO in certain situations have been suggested. For example, semicarbazide-sensitive amine oxidases (SSAOs) can turn aminoacetone and specific biogenic amines into MGO and hydrogen peroxide [15]. In addition, also triose phosphate isomerase (TPI) exhibits collateral MGO-producing activities, as supported by studies on TPI deficiency syndrome [16]. Furthermore, several studies have suggested the expression of MGS-like activity in specific human tissues. Among these, Prantner et al. reported increased MGO production during M1 macrophages activation and hypothesized its role as a signalling molecule [17]. Overall, compared with the non-enzymatic via, enzymatic MGO formation likely represents a quantitatively minor contribution in human physiology.

### 3.1. Methylglyoxal and Its Adducts

In protein systems, MGO preferentially reacts with arginine residues to form hydroimidazolones, the most prevalent of which is MG-H1 [Nδ-(5-hydro-5-methyl-4-imidazolon-2-yl)-ornithine], and to a lesser extent with lysine residues to generate CEL [Nϵ-(1-carboxyethyl) lysine] [18]. Proteins showing increased MGO-derived adduct formation are collectively referred to as dicarbonyl proteome (DCP) and include histones, albumin, hemoglobin, some extracellular matrix proteins, and others [19]. In general, alterations affecting proteins are tightly associated with cytotoxicity [20]. In particular, the increased formation of MG-H1 resulting from reduced activity of the glyoxalase 1 (GLO1) or decreased renal clearance leads to protein dysfunction, age-related functional decline, and exacerbation of pathological conditions [21]. Ahmed et al. elucidated the susceptibility of human serum albumin to MGO modification and identified several glycation hotspots associated with functional impairment and altered drug-binding capacity [22]. Other studies have demonstrated the interaction between MGO and collagen IV of the vascular basement membrane, particularly at the integrin binding sites promoting endothelial cell detachment, cell death, and inhibition of angiogenesis [23]. MGO also induces modifications in hemoglobin that promote iron release from the heme group, thereby increasing the risk of reactive oxygen species formation and oxidative cellular damage [24].

MGO preferentially binds to DNA guanosine residues, yielding various adducts, among which, imidazopurinone (MGdG) represents the most abundant species, followed to a lesser degree by CEdG [N2-(1-carboxyethyl)-2′-deoxyguanosine] [25]. Early studies demonstrated the mutagenic potential of MGO-derived DNA modifications [26]. In *S. cerevisiae*, the mutagenic activity of MGO has also been associated with interference in DNA repair and replication mechanisms [27]. Beyond its mutagenic effect, MGO adducts also participate in the formation of DNA–protein cross-links, that slow the replication fork progression and promote chromosomal instability [28].

### 3.2. Dicarbonyl Stress and Methylglyoxal

Reactive Carbonyl Species (RCS) are a class of electrophilic molecules characterized by the presence of highly reactive carbonyl groups, among which MGO represents the quantitatively and physio-pathologically most relevant compound [29]. These molecules can be generated both endogenously, as by-products of cellular metabolism, and exogenously through processes such as the Maillard reaction. RCS constitute the main precursors of AGEs, which, as mentioned above, are a heterogeneous and harmful group of compounds formed through the non-enzymatic modification of proteins, lipids, and nucleic acids [30].

Dicarbonyl stress is triggered when the balance between pro-glycating agents, such as MGO, and anti-glycation enzymatic defenses shifts toward the former. Much like oxidative stress, baseline concentrations of reactive dicarbonyls might serve physiological signaling purposes, yet their over-accumulation leads to harmful effects. This idea was first introduced as a pathological process associated with natural aging and conditions like diabetes, kidney failure, and Alzheimer’s [31]. Specifically, dicarbonyl stress refers to the accumulation of reactive dicarbonyl compounds that induces alterations in function and structure of proteins and DNA, ultimately contributing to worsening or onset of cellular and tissue dysfunction [32]. Therefore, the relationship between RCS, dicarbonyl stress, and AGEs can be described as a direct cause–effect pathway, in which RCS and MGO in particular, represent the chemical mediators through which metabolic stress is translated into molecular damage.

### 3.3. Methylglyoxal Clearance Systems

**Figure 1:**
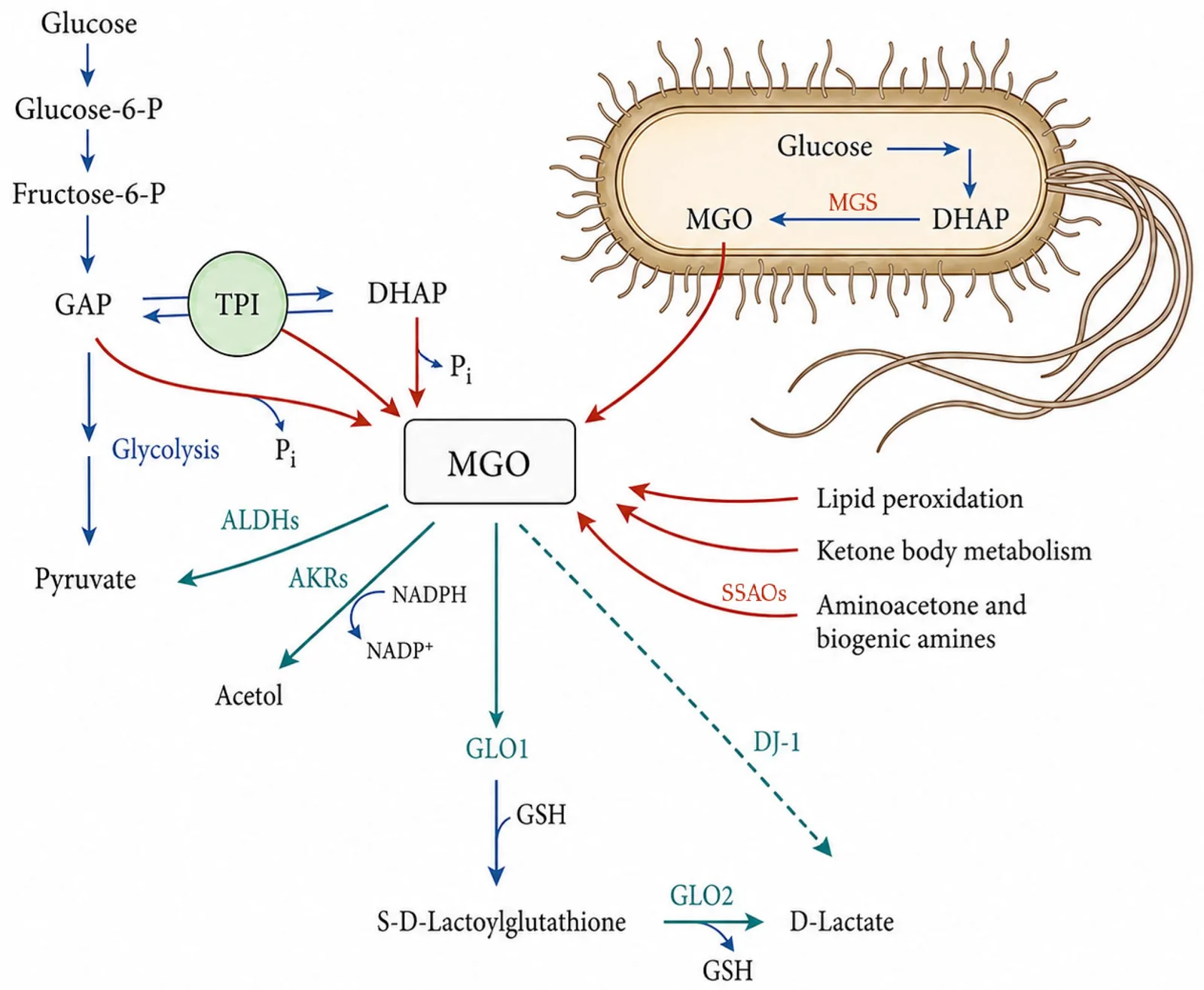
Overview of MGO production and clearance pathways. Red arrows indicate production routes, teal arrows indicate clearance pathways, and dashed arrows denote putative mechanisms. MGO is generated mainly through spontaneous P_i_ elimination from GAP and DHAP, with additional contributions from TPI side activity, bacterial MGS, lipid peroxidation, ketone body metabolism, and SSAOs. Clearance is mediated by the glyoxalase system, ALDHs, AKRs, and DJ-1.

MGO homeostasis is primarily regulated by the glutathione-dependent glyoxalase pathway. This ubiquitous pathway consists of two sequential enzymatic steps. GLO1 catalyzes the conversion of MGO and reduced glutathione (GSH) to S-D-lactoylglutathione, which is subsequently hydrolysed by glyoxalase 2 (GLO2) to produce D-lactate, while simultaneously regenerating GSH [2]. Consequently, reduced GLO1 activity or depletion of intracellular GSH promotes MGO accumulation and favors the development of dicarbonyl stress [33]. In addition to the GLO1 pathway, alternative glutathione-independent routes have been described, including those mediated by aldo-keto reductases (AKRs) and aldehyde dehydrogenases (ALDHs). AKRs are NADPH-dependent enzymes that reduces MGO to acetol, whereas ALDHs oxidize MGO to pyruvate [34]. Although quantitatively less prominent, these pathways are metabolically significant because they directly convert a toxic metabolite to pyruvate, a central intermediate in energy metabolism. A more recent mechanism involved in the MGO elimination is driven by DJ-1, also known as PARK7. The latter exhibits scavenging properties, yet its specific mechanism of action remains debated. Some studies suggest that DJ-1 directly metabolizes MGO to lactate, while others propose that it functions as a deglycase, removing MGO-derived adducts from proteins and DNA [35]. Overall, while the glyoxalase system accounts for the majority of MGO clearance, these alternative pathways may become crucial under conditions of dicarbonyl stress or when the primary via is impaired, as occurs during ageing, diabetes and neurodegenerative disorders (Figure 1).

### 3.4. Tissue Distribution and MGO in Intestinal Lumen

While endogenous glycolytic metabolism is the primary driver of intracellular MGO production, the total dicarbonyl burden is also influenced by exogenous intake. Diets rich in highly processed foods contain substantial amounts of pre-formed MGO and dietary AGEs generated during heat treatments [36]. However, while dietary AGEs can be partially absorbed and retained, contributing to the systemic AGE pool, the ingestion of free MGO itself is unlikely to substantially affect circulating free MGO concentrations. This is because luminal MGO is extensively metabolized or reacts with macromolecules within the gastrointestinal tract prior to absorption [7,37].

Consequently, the levels of MGO throughout the body are largely defined by how well endogenous production is balanced against clearance rates, while nutrition may exert an indirect effect on dicarbonyl stress by influencing factors such as metabolism, inflammation, the strength of the gut barrier, and redox stability. The intestinal epithelium functions as an essential physical and biochemical gatekeeper when it encounters dietary dicarbonyls and their precursors at the gastrointestinal interface. At the same time, the capture or secretion of both consumed and internally produced dicarbonyls within the intestinal space establishes a specialized and evolving microenvironment.

This luminal free MGO pool is the primary site of interaction between host-derived or dietary dicarbonyls and the gut microbiota. Emerging evidence suggest that intestinal microbes harbor enzymatic activities capable of both producing MGO (e.g., via MGS) and degrading it through bacterial glyoxalase systems [38]. These findings indicate that the microbiota may actively contribute to local intestinal carbonyl metabolism and modulate host susceptibility to dicarbonyl stress via its effects on redox status and barrier integrity [11]. Furthermore, the continuous exposure of the microbiota to luminal MGO can exert selective pressure, potentially inhibiting the growth of susceptible beneficial taxa while favouring more resilient pathobionts [39]. The disruption of the balance between endogenous production, exogenous intake, and the microbiota-mediated removal of MGO in gut lumen can ultimately compromise barrier integrity, triggering the inflammatory cascades associated with metabolic dysfunction [40]. Understanding these interactions requires a comprehensive evaluation of the composition and functional evolution of microbiota, which are central to host physiology across the lifespan.

## 4. Gut Microbiota Dysbiosis, Intestinal Permeability, and Systemic Inflammation

The human gastrointestinal tract harbours a complex and dynamic community of microorganisms, collectively termed the gut microbiota (GM), comprising bacteria, archaea, viruses and fungi [5]. Because of its broad taxonomic diversity and functional potential, the GM effectively serves as an essential metabolically active organ for the host’s physiological health. Its roles include managing nutrient metabolism, modulating the immune response, and preserving homeostasis within the gut [41].

During the process of physiological ageing, fluctuations in key microbial metabolites (e.g., butyrate, acetate, lactate, succinate and propionate) cause intestinal dysbiosis, a hallmark of ageing [6,42]. These compositional and functional changes are consistently associated with chronic inflammaging and metabolic dysfunction in older adults [42,43]. It is significant that the inflammatory impacts of dysbiosis linked to aging vary considerably from person to person, implying that personal host factors actively regulate the connection between microbial diversity and immune activity throughout the aging process [44,45].

Age-related dysbiosis often presents as increased intestinal permeability alongside persistent, low-grade systemic inflammation. These features directly overlap with the hallmarks of immunosenescence, thereby increasing the risk of chronic inflammatory and metabolic disorders [42,45,46]. From a mechanistic perspective, a compromised intestinal barrier enables luminal microbial components, such as lipopolysaccharide (LPS), to translocate into the systemic circulation, thereby promoting sustained activation of innate immune pathways [40]. This translocation is the main cause of systemic inflammation in elderly people [47,48,49].

**Figure 2:**
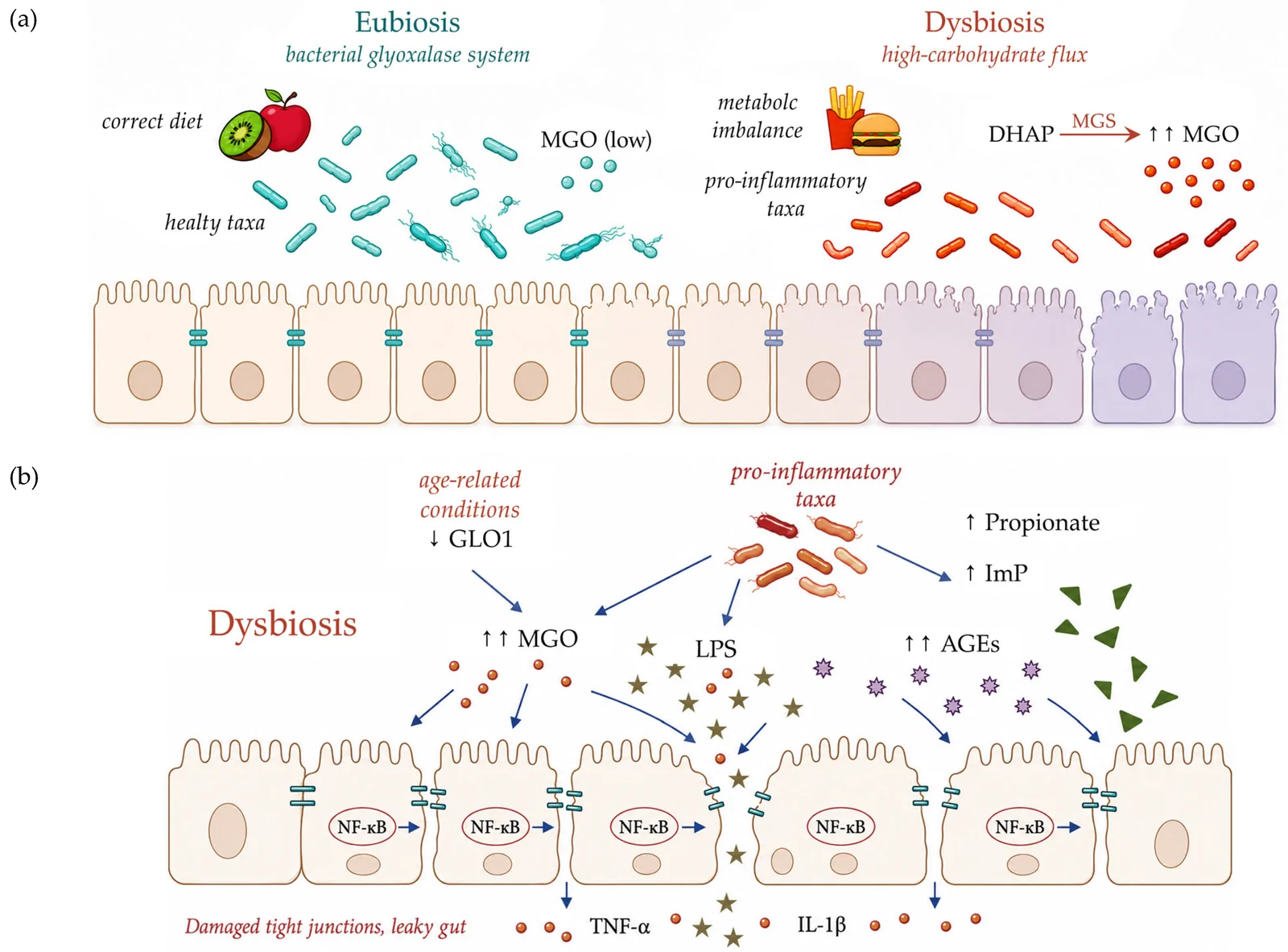
Proposed links among age-related gut dysbiosis, methylglyoxal (MGO) metabolism, and intestinal barrier dysfunction. (**a**) Dysbiosis and increased carbohydrate flux may enhance microbial MGO production through MGS while reducing neutralization capacity. (**b**) MGO, AGEs, LPS, and altered microbial metabolites may impair tight junctions, activate NF-κB signaling, and promote inflammation. Reduced GLO1 activity may further increase MGO accumulation, sustaining a putative feedback loop between dysbiosis, carbonyl stress, and intestinal permeability.

Persistent low-grade inflammation is linked to metabolic functions, where it interferes with insulin signaling and glucose balance while triggering stress within cells. This constant state of immune activation shifts cellular energy patterns, possibly promoting the collection of metabolic by-products and intensifying general metabolic instability [7,50]. Rather than acting as an isolated causal determinant, dysbiosis functions within a reciprocal host-microbiota network in which inflammatory tone, barrier integrity and metabolic fluxes reinforce each other. In this context, metabolic by-products from the host and microbiota are critical mediators that link inflammation to metabolic dysfunction, especially when glycolytic activity is disrupted [51,52,53] (Figure 2a).

### 4.1. Methylglyoxal, AGEs, and the Microbiota–Metabolism–Inflammation Axis

Systemic MGO levels are mainly determined by endogenous production and clearance, whereas diet and the gut microbiota may influence dicarbonyl stress indirectly through metabolic, inflammatory, and intestinal barrier mechanisms [11,54,55]. Although dietary MGO and its precursors could theoretically contribute to systemic dicarbonyl stress, their direct impact on circulating MGO levels remains uncertain [56].

In the intestinal ecosystem, microbial metabolites derived from dietary fibre and amino acids act as vital signalling molecules that regulate host-microbiota communication. A notable example is propionate, a short-chain fatty acid produced through fibre fermentation by specific bacteria such as *Bacteroides*, *Prevotella* and *Veillonella* [57]. Propionate has multifaceted effects: it provides an essential energy source for the colonic epithelium and regulates immune and metabolic functions. However, emerging evidence suggests that its physiological outcomes are highly context-dependent, varying according to dosage, tissue localisation and the host’s underlying metabolic state [58,59,60,61].

Under insulin-resistant conditions, increased glycolytic pressure may enhance endogenous MGO formation, while age-related impairment of GLO1 may reduce its enzymatic inactivation. The resulting accumulation of free MGO and endogenous MGO-derived AGEs may amplify oxidative and inflammatory signaling and further impair glucose homeostasis [50,62,63,64,65]. Consequently, in vivo models of GLO1 knockdown demonstrate age-related impairments in glucose tolerance and beta-cell function, alongside heightened pancreatic inflammation [50,66].

Intestinal bacteria can both produce and neutralize MGO. Under conditions of high carbohydrate availability or metabolic imbalance, bacterial MGO production may increase, whereas dysbiosis may impair glyoxalase-mediated clearance, favouring luminal MGO accumulation, AGE formation, and epithelial barrier dysfunction [38,39,67,68,69,70]. Elevated luminal MGO may activate NF-κB signalling and increase the expression of TNF-α and IL-1β, thereby aggravating epithelial barrier dysfunction and intestinal inflammation [38,68,69,70] (Figure 2b).

Taken together, these observations suggest that age-related dysbiosis interacts dynamically with systemic metabolic and inflammatory pathways. When intestinal permeability is compromised, altered microbial metabolite profiles, including propionate and imidazole propionate (ImP), impair glucose regulation. This creates a self-reinforcing loop that accelerates MGO formation and total AGE burden increase [40]. In this integrative model, MGO does not act as a primary trigger. Instead, it acts as a key consequence of metabolic dysregulation and a critical pathogenic mediator that links carbonyl stress to inflammatory processes, insulin resistance and age-related metabolic disorders such as type 2 diabetes mellitus (T2DM) [7,50,66].

### 4.2. In Vivo and In Vitro Studies Linking MGO and GM

Emerging preclinical evidence suggests that MGO plays a crucial role in modulating the composition and function of the intestinal microbiota. According to Morresi et al., MGO exposure affects the integrity of the intestinal barrier in cell line models. Their study also showed that MGO induces apoptosis alongside elevated mitochondrial and cytosolic reactive oxygen species. Furthermore, the oxidative stress caused by MGO was tied to the activation of the NF-κB signaling pathway, along with increased production of proinflammatory cytokines such as TNF-α and antioxidant defenses like SOD1 and catalase. The increased expression of nuclear proteins γH2AX (phosphorylated form of the histone variant H) indicated DNA damage in MGO-treated cells. Additionally, enzymes that govern chromatin remodelling such as Histone Deacetylase 1 (HDAC1) and Histone Deacetylase 2 (HDAC2) was observed a decrease in expression, consistent with increased acetylation of histone H4, suggesting a potential boosting activity of MGO on histone acetylation processes. Furthermore, in MGO-treated cells, inhibition of the expression of Ten-eleven translocation (TET) proteins was observed, particularly of TET1 and TET2, key epigenetic enzymes, corroborating the hypothesis of a potential impact of MGO on DNA methylation homeostasis [68].

In a study involving Sprague–Dawley rats, Qu et al. showed that dietary AGEs led to changes in cecal microbiota and metabolites, as well as altered colon permeability. The researchers observed that AGE treatment decreased the diversity and abundance of microbial life, particularly SCFA-producing bacteria such as Ruminococcaceae and *Allo-prevotella*, while increasing the prevalence of potentially harmful microbes like *Desulfovibrio* and *Bacteroides*, which could negatively affect the health of the host [71].

As previously mentioned, MGO has emerged as a significant precursor implicated in the pathogenesis of type 2 diabetes and neurodegenerative diseases. Tirelli et al. investigated the impact of prolonged oral administration of free MGO on gut health in an aged murine model (B6/129PF2 mice, 18–20 months). Their analysis demonstrated that chronic exposure to this reactive dicarbonyl compound induces significant dysbiosis, characterized by an altered Firmicutes-to-Bacteroidetes ratio and an adaptive increase in genera such as Lachnospiraceae and Akkermansia genera.

Simultaneously, a notable decline in bacteria that generate short-chain fatty acids (SCFAs), such as the Harryflintia, Intestinimonas, and Ruminococcaceae genera, impairs metabolic routes in the lumen and modifies intestinal permeability by impacting the regulation of tight junction proteins like zonulin-1 and occludin [70]. The evidence is summarized in Table 1.

## 5. MGO and Cellular Senescence

At the cellular level, MGO and MGO-derived AGEs contribute to tissue dysfunction through multiple mechanisms. These include receptor-independent effects, such as the direct covalent modification and inactivation of proteins, mitochondrial dysfunction, and redox imbalance, as well as receptor-mediated pathways involving activation of the RAGE and downstream pro-inflammatory signalling cascades, including NF-κB activation [73]. Collectively, these processes promote chronic inflammation, endothelial dysfunction, and progressive tissue damage, thereby contributing to ageing-related cellular decline [74].

Cellular senescence, a hallmark of ageing, is characterized by stable cell-cycle arrest accompanied by metabolic alterations and the acquisition of a senescence-associated secretory phenotype (SASP), which contributes to chronic low-grade inflammation and dysfunction of the surrounding tissue microenvironment [75]. Although the extent to which MGO induces bona fide cellular senescence remains incompletely understood, accumulating evidence suggests that dicarbonyl stress may activate several pathways associated with the acquisition of the senescent phenotype [31].

One of the earliest studies supporting this hypothesis was conducted by Sejersen and Rattan, who demonstrated that exposure of human fibroblasts to MGO and glyoxal induced marked morphological alterations consistent with cellular senescence. MGO-treated cells exhibited irreversible cell-cycle arrest, increased intracellular H_2_O_2_ levels, altered activities of antioxidant enzymes such as superoxide dismutase and catalase, and increased expression of several senescence-associated biomarkers [4].

More recently, MGO accumulation has been shown to promote endothelial senescence through activation of the integrated stress response (ISR). Experimental evidence obtained in human umbilical vein endothelial cells (HUVECs) and in multiple murine ageing models demonstrated that MGO induces the dimerization and autophosphorylation of Eukaryotic Translation Initiation Factor 2 Alpha Kinase 2 (EIF2AK2, also known as PKR), triggering the downstream eIF2α/ATF4/CHOP signalling cascade. Activation of this pathway promotes endothelial injury by increasing the expression of canonical senescence regulators, including p53, p21, and p16, while simultaneously enhancing the expression of pro-apoptotic mediators such as cleaved caspase-3 and Bax and suppressing the anti-apoptotic protein Bcl-2. Interestingly, naturally aged mice exhibited a distinct molecular signature characterized by persistent EIF2AK2/eIF2α activation and CHOP induction despite reduced activating transcription factor 4, also known as ATF4, expression, a phenomenon attributed to age-related ribosomal depletion. In contrast, exogenous MGO administration preserved canonical ATF4 signalling without affecting ribosome abundance. Importantly, pharmacological inhibition of EIF2AK2 using C16, as well as treatment with berberine, which directly interferes with EIF2AK2 dimerization and autophosphorylation, significantly attenuated MGO-induced endothelial senescence and apoptosis [76]. These findings identify EIF2AK2 as a potential therapeutic target linking dicarbonyl stress to vascular ageing.

## 6. Pathophysiological Implications of the MGO–Microbiota Axis

Accumulating evidence indicates that the interaction between MGO and the gut microbiota extends beyond a purely biochemical association and may have broad clinical relevance across multiple chronic disorders (Table 2). Dietary exposure to AGEs can remodel gut microbial communities and promote inflammatory disturbances [77]. In parallel, MGO is increasingly recognized as a reactive carbonyl mediator involved in oxidative stress, AGE formation, and tissue dysfunction across diverse pathological states [78]. Emerging data further link MGO-related carbonyl stress to obesity, insulin resistance, and metabolic dysfunction-associated steatotic liver disease, supporting its involvement in gut–metabolic organ crosstalk.

### 6.1. Metabolic and Cardiometabolic Dysfunction

The pathological relevance of the MGO–microbiota axis is most evident in metabolic disorders characterized by nutrient excess, insulin resistance, and chronic low-grade inflammation. In these settings, increased glycolytic flux, mitochondrial dysfunction, and impaired antioxidant defenses favor endogenous MGO accumulation, while dietary AGEs may further increase systemic carbonyl burden. Concurrently, obesity-associated dysbiosis can impair host metabolic regulation and promote glucose intolerance, lipid overload, and inflammation.

Findings from experimental research show that diets high in AGEs might trigger gut dysbiosis and metabolic impairment. Specifically in mice, prolonged dietary intake of AGEs correlates with shifts in microbiome makeup, hindered insulin signaling, decreased production of GIP and GLP-1 (incretin hormones), and an uptick in inflammatory agents like IL-1β and IL-17 [79]. High-AGE diets have also been linked to insulin resistance and glucose dysregulation, although reported taxonomic shifts differ across experimental models [80]. In the study of Wang et al. fed mice with high-AGE diets showed reduced abundance of butyrate-producing taxa, such as Bacteroidales_S24-7, Ruminococcaceae, and Lachnospiraceae, and the analysis of gut microbiota revealed significantly enriched fructose and mannose metabolism. The authors hypothesized that the loss of these beneficial taxa could lead to a deterioration of intestinal barrier integrity and a state of chronic low-grade inflammation [81].

**Figure 3:**
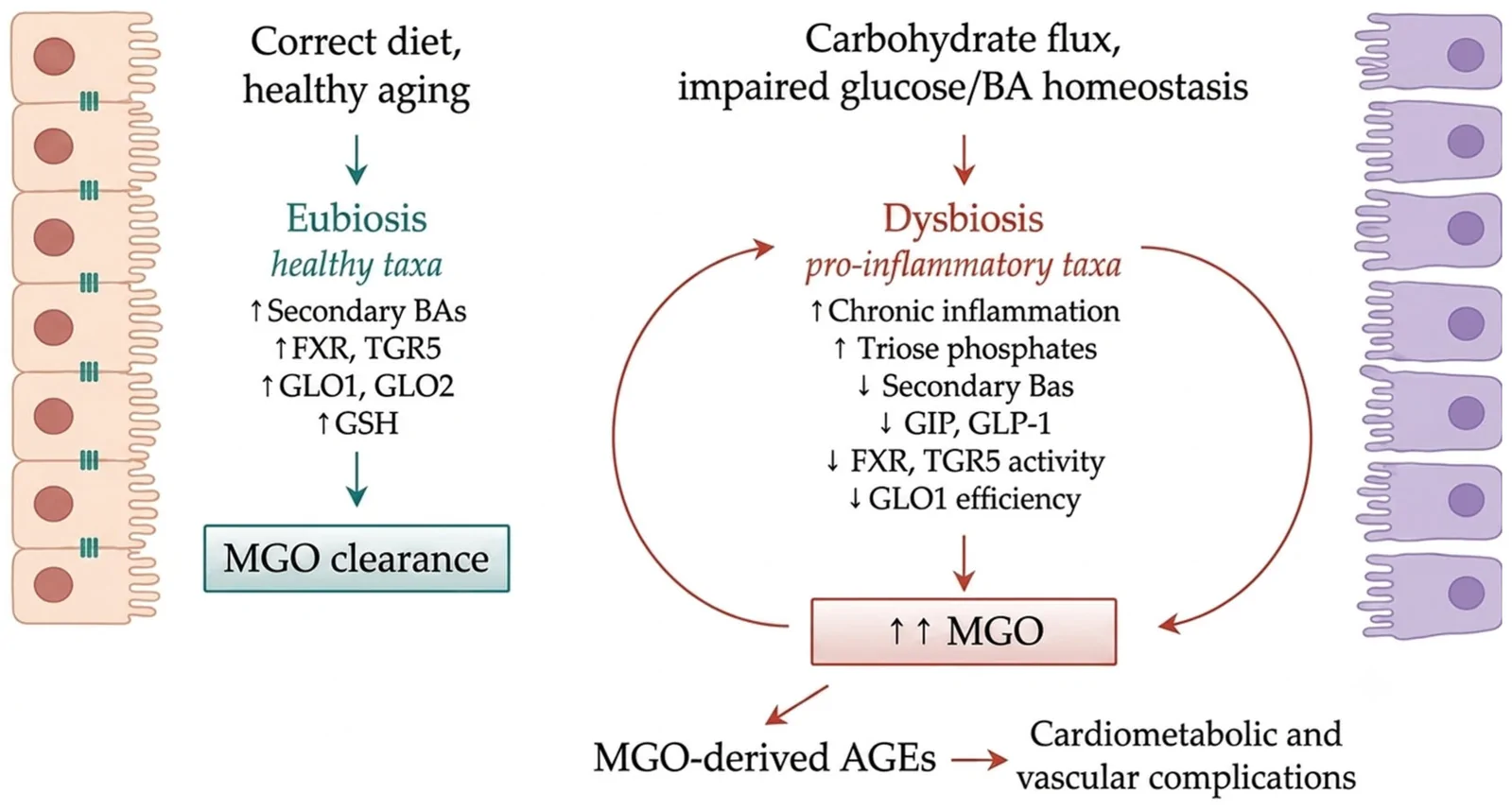
In eubiotic conditions, beneficial microbial communities contribute to intestinal homeostasis and may support MGO clearance. In contrast, dysbiosis, characterized by the expansion of pro-inflammatory taxa and the loss of protective microbial functions, may promote intestinal inflammation, barrier dysfunction, and systemic metabolic stress. Increased MGO accumulation, driven by enhanced glycolytic flux, dietary AGEs, oxidative stress, and impaired enzymatic inactivation, further amplifies dysbiosis and inflammatory signalling. This self-reinforcing MGO–microbiota axis may contribute to MASLD, adipose dysfunction, vascular injury, and increased cardiometabolic risk.

Bile acid signalling may provide an additional mechanistic link between dysbiosis and host metabolism. Microbiota-dependent remodelling of the bile acid pool modulates farnesoid X receptor (FXR) and Takeda G protein-coupled receptor 5 (TGR5) signalling, thereby influencing glucose homeostasis, insulin sensitivity, and adipose function. Consistently, the bile acid tauroursodeoxycholic acid (TUDCA), the taurine conjugated form of the ursodeoxycholic acid (UDCA), has been associated with changes in gut microbial composition, while bacterially modified bile acids can promote white adipose tissue browning through FXR signalling, linking microbiota-derived bile acid metabolism to energy balance [82,83]. FXR acts as a metabolic sensor, suppressing hepatic gluconeogenesis and modulating glycolysis. Under conditions of metabolic overload, accelerated glycolysis drives a proportional increase in endogenous MGO production. Physiological FXR activation curbs the glycemic burden by dampening excessive glucose fluxes and suppressing hepatic glucose production, theoretically establishing an upper limit for the intracellular generation of toxic dicarbonyl species [84]. Moreover, AGEs derived from endogenous MGO alter the composition of the intestinal microbiota, which subsequently disrupts the deconjugation and conversion of primary bile acids into secondary bile acids [85]. From a molecular perspective, intense dicarbonyl stress compromises the operation of nuclear receptors by way of MGO-induced glycation, which alters key amino acid residues in the binding domains of FXR or destabilizes its heterodimer partner, RXR. This post-translational change reduces the sensitivity of FXR, causing liver cells to experience continuous inflammation and disrupted lipid processing [86]. FXR agonists stimulate pathways that indirectly sustain or upregulate GLO1/GLO2 expression and boost GSH synthesis, including those linked to nuclear factor erythroid 2-related factor 2 (Nrf2). Active bile acid signalling facilitates the clearance of MGO, thereby shielding hepatic parenchymal cells and endothelial structures from tissue injury and macrovascular damage [87,88]. Integrating these findings into the biology of ageing reveals a potential pathological loop that drives metabolic decline in elderly populations. As tissues age, the transcriptional efficiency of FXR and the enzymatic capacity of GLO1 both decline progressively. The loss of functional FXR results in impaired glucose handling and increased glycolytic flux, leading to elevated MGO production. Widespread dicarbonyl stress is caused by the collection of MGO, leading to tissue senescence and extracellular matrix cross-linking, alongside the disruption of the gut-liver axis and the further weakening of FXR activity. Consequently, targeting FXR offers a hopeful therapeutic path; employing selective FXR agonists may halt this cycle, mitigating dicarbonyl stress and protecting metabolic vigor as one ages. Furthermore, variations in this axis might be pertinent to MASLD, where the progression of the disease may be driven by carbonyl stress associated with MGO [87] (Figure 3).

Clinical observations further support the systemic relevance of MGO-related carbonyl stress. Elevated plasma MGO has been associated with cardiovascular events in type 1 diabetes and poorer outcomes in severe limb ischemia, while MGO-derived AGEs in carotid plaques correlate with inflammation and plaque vulnerability [89,90,91]. In parallel, the microbiota-related metabolite trimethylamine N-oxide (TMAO) has been linked to aortic stiffness and higher systolic blood pressure through mechanisms involving AGE accumulation and oxidative stress, both in humans and mice [92].

Human intervention data are limited to specific populations and remain largely biomarker-based, without established causal links to MGO-related clinical outcomes. In pharmacological studies, metformin was associated with attenuated carbonyl stress in type 2 diabetes, with lower plasma MGO despite comparable glycemic control, while a 24-week prospective study showed reduced plasma MGO and Nε-(carboxymethyl)lysine-modified proteins together with increased GLO1 activity [72,93]. Short-term benfotiamine prevented AGE-rich meal-induced postprandial increases in total serum MGO-derived adducts and carboxymethyllysine-reactive AGEs, as well as oxidative stress and endothelial dysfunction [94]. Pyridoxamine reduced plasma MGO, protein-bound MGO-derived MG-H1, and soluble endothelial adhesion markers in abdominally obese adults, without improving insulin sensitivity or vascular function [95]. Dietary and supplement interventions have also been investigated for their potential to modulate MGO-related carbonyl stress. In this regard, long-term adherence to a Mediterranean diet in patients with type 2 diabetes and coronary heart disease was associated with reduced circulating MGO-related AGE levels and increased GLO1 expression; in participants with mildly decreased baseline estimated glomerular filtration rate (eGFR), these changes were accompanied by a slower decline in renal function [96]. Interestingly, these findings are consistent with a possible gut–kidney extension of the proposed MGO–AGE–microbiota feedback loop, in which depletion of short-chain fatty acid (SCFA)-producing bacteria may impair intestinal barrier integrity, favor the accumulation of gut-derived microbial toxins, and contribute to systemic inflammation and vascular injury, particularly when impaired renal function further increases the systemic burden of glycated products [97]. Shorter randomized trials reported reductions in circulating MGO with quercetin 3-glucoside in apparently healthy (pre)hypertensive adults, and with trans-resveratrol/hesperetin or pyridoxamine in overweight or obese participants [95,98,99].

Collectively, these findings suggest that MGO operates within a metabolic network in which diet, dysbiosis, altered bile acid signalling, adipose dysfunction, and vascular injury converge to influence carbonyl stress and cardiometabolic risk.

### 6.2. Intestinal Barrier Dysfunction and Gastrointestinal Disease

The gastrointestinal tract serves as a crucial point of contact for food-derived glycation products, metabolic activities of microbes, and the body’s own reactions. In this setting, the connection between methylglyoxal and gut flora potentially affects digestive stability by altering bacterial makeup, the integrity of the gut lining, and the synthesis of metabolites in the intestinal cavity. Experimental evidence indicates that glycated dietary proteins can modify the colonic microbiota toward a less favorable profile. In in vitro colonic models inoculated with faecal microbiota from ulcerative colitis patients and non-ulcerative volunteers, glycated bovine serum albumin (BSA) reduced *Bifidobacteria* and *Eubacteria* while increasing *Clostridia*, *Bacteroides*, and sulfate-reducing bacteria, with parallel reductions in short-chain fatty acids [100]. Dietary AGEs may also impair gut barrier function. In diabetic mice, a high-AGE diet increased intestinal permeability, altered the Firmicutes/Bacteroidetes ratio, and worsened albuminuria [101].

MGO itself may also arise locally from bacterial carbohydrate metabolism. Experimental work has proposed that microbial generation of MGO and related metabolites from incompletely absorbed carbohydrates could influence bacterial growth and contribute to gastrointestinal symptoms, including those observed in irritable bowel syndrome [102].

Overall, current evidence supports the intestine as both a source and a target of glycation-related stress, linking diet, microbial imbalance, and altered gut homeostasis.

### 6.3. MGO, Frailty and Functional Decline

The nervous system may be particularly susceptible to MGO-induced damage because of its high energy demand and limited regenerative capacity. Central neurons may limit MGO production by preferentially using astrocyte-derived lactate, which is converted into pyruvate and acetyl-CoA, thereby bypassing part of the glycolytic pathway responsible for MGO generation [62]. By contrast, peripheral neurons appear to lack comparable protective mechanisms, and MGO-mediated modification of the Nav1.8 sodium channel has been implicated in enhanced nociceptor excitability and hyperalgesia in diabetic neuropathy [103].

Evidence linking MGO to brain ageing is derived predominantly from preclinical models. In *Caenorhabditis elegans*, MGO produced by intestinal *Escherichia coli* accelerated ageing through modulation of the TORC2/SGK-1/DAF-16 pathway [104]. In D-galactose-induced ageing mice, cortical MGO accumulation was associated with cognitive impairment, reduced glyoxalase activity, and increased neuroinflammatory signalling [105].

Similarly, fecal microbiota transplantation from aged to young rats impaired cognition and increased cerebral AGEs, RAGE, inflammatory cytokines, and oxidative stress [106]. In the microglia of aged mice, age-related microbiota-dependent accumulation of the N(6)-carboxymethyllysine AGE has also been shown to promote oxidative stress, mitochondrial dysfunction, and ATP depletion [107].

Human evidence remains limited, with intervention data largely biomarker-based and observational data mostly associative. In apparently healthy elderly subjects, a short randomized cross-over trial reported reduced plasma MGO after citrus/pomegranate supplementation, although no clinical outcomes linked to this reduction were assessed [108].

Immunohistochemical studies have shown AGE colocalization with astrocytes and microglial cells in Alzheimer’s disease brains, although direct visualization of MGO accumulation at single-cell resolution is currently unavailable [109]. MGO may also interact with receptors and ion channels and modify neurotransmitters, potentially affecting neuronal excitability and behavior [110]. However, these mechanistic observations do not establish a direct causal role for the MGO–microbiota axis in human neurodegeneration.

Other microbiota-derived mediators may act in parallel. TMAO increases with age in humans and mice and has been associated with poorer cognitive performance, neuroinflammation, and astrocyte activation [111]. In Parkinson’s disease, enrichment of *Desulfovibrio* has been associated with greater disease severity, and bacterial products such as hydrogen sulfide, lipopolysaccharide, and magnetite have been proposed as potential contributors to α-synuclein aggregation [112]. These findings support microbiota-related mechanisms of neurological dysfunction but do not provide direct evidence of MGO-mediated causality.

Skeletal muscle may be another target of carbonyl stress. In myoblast cells, MGO induces oxidative stress, inflammatory activation, mitochondrial abnormalities, reduced tricarboxylic acid cycle (TCA) intermediates, ATP depletion, and metabolic rerouting toward fatty acid synthesis [8]. In parallel, MGO-derived AGEs impair myogenic differentiation by increasing AGE–RAGE signalling and reducing myogenic differentiation 1 (MYOD) and myogenin (MYOG), thereby limiting myotube formation [113].

Overall, current evidence supports a contributory role for dysbiosis, AGEs, MGO-related carbonyl stress, and microbiota-derived metabolites in age-associated functional decline. However, most available data derive from in vitro or animal models, and the direct contribution of gut-derived MGO to brain ageing remains uncertain. Indirect effects mediated by changes in insulin sensitivity, systemic glucose metabolism, and inflammatory signaling are biologically plausible but require further experimental and clinical validation.

**Table 2:** Evidence from studies on the MGO–microbiota axis across different diseases.

Pathological Condition	Role of Microbiota/Dysbiosis	Contribution of MGO/AGEs	Proposed Mechanisms	References
Obesity and insulin resistance	Dysbiosis impairs metabolic regulation and promotes inflammation	Increased endogenous MGO production secondary to enhanced glycolytic flux and metabolic stress	Chronic inflammation, impaired insulin signalling, oxidative stress, AGE accumulation	[79,80,81]
Type 2 diabetes mellitus	Altered microbiota-derived metabolites (e.g., ImP, SCFAs) contribute to insulin resistance	Elevated MGO promotes dicarbonyl stress and AGE formation	Hyperglycemia → increased triose phosphates → MGO accumulation → metabolic dysfunction	[7,50]
Metabolic dysfunction-associated steatotic liver disease	Dysbiosis alters bile acid metabolism and metabolic homeostasis	MGO-derived carbonyl stress may contribute to disease progression	FXR/TGR5 dysregulation, oxidative stress, inflammation	[82,87]
Cardiovascular disease	Microbiota-derived metabolites (e.g., TMAO) promote vascular dysfunction	Elevated circulating MGO and MGO-derived AGEs correlate with vascular injury	Endothelial dysfunction, oxidative stress, plaque instability, inflammation	[89,90,91,92]
Intestinal barrier dysfunction (“leaky gut”)	Dysbiosis alters microbial composition and reduces SCFA production	MGO and AGEs impair epithelial integrity	Tight junction disruption, inflammatory signalling, altered microbiota composition	[100,101]
Inflammatory bowel disorders/IBS	Bacterial carbohydrate fermentation generates reactive metabolites	Local MGO production may contribute to intestinal symptoms	Luminal carbonyl stress, altered bacterial growth, inflammation	[102]
Brain ageing and cognitive decline	Aged microbiota promotes neuroinflammation and AGE accumulation	MGO-derived AGEs contribute to oxidative stress and neuronal dysfunction	RAGE activation, ROS production, mitochondrial dysfunction	[106,107]
Frailty and sarcopenia	Dysbiosis may reduce beneficial microbial metabolites involved in muscle homeostasis	MGO impairs muscle cell metabolism and differentiation	Mitochondrial dysfunction, ATP depletion, increased AGE–RAGE signalling, reduced MYOD/MYOG expression	[8,113]
Neurodegenerative diseases	Altered microbial composition (e.g., Desulfovibrio enrichment) may promote neurodegeneration	MGO-related carbonyl stress may exacerbate neuronal vulnerability	Neuroinflammation, oxidative stress, α-synuclein aggregation, mitochondrial dysfunction	[111,112]
Age-related functional decline	Reduced microbial diversity and altered metabolite production increase systemic inflammation	MGO acts as a mediator of carbonyl stress rather than a sole causal factor	Inflammaging, oxidative stress, tissue dysfunction, metabolic dysregulation	[7,50]

## 7. Conclusions

The investigation of MGO reveals a complex network of interactions between metabolic pathways, microbial ecosystems and systemic health outcomes. As a key dicarbonyl metabolite in the formation of AGEs, MGO has been linked in preclinical and observational studies to the pathophysiology of metabolic disorders and chronic inflammatory conditions. While data suggest that the gut microbiota may exert a bidirectional regulatory influence on MGO homeostasis by producing and metabolising this reactive compound, these insights largely derive from heterogeneous experimental models and direct human evidence remains scarce.

The current literature outlines associations between dysbiosis, altered metabolic control and elevated MGO accumulation. However, the exact causal relationship underlying these associations has yet to be fully resolved. It is unclear whether microbial shifts act as a primary driver or a secondary consequence of MGO-mediated tissue injury and systemic inflammation, but this is a critical open question with significant implications for metabolic syndrome, cardiovascular disease, and neurodegenerative pathologies.

Consequently, although targeting microbiota restoration or enhancing MGO disposal pathways are compelling theoretical approaches to attenuating pathological effects, there are currently no validated MGO- or microbiota-targeted clinical interventions. By filling a significant gap in the literature, this review delineates how the intersection of dicarbonyl stress and intestinal dysbiosis accelerates senescence. Future research should transition from diverse, observational and preclinical frameworks to standardised, translational studies. Validating these mechanistic pathways in human cohorts will be essential to establishing a definitive framework that integrates microbial and metabolic interactions within human physiology, and to safely evaluating potential interventional strategies during ageing.

## Figures and Tables

**Table 1:** Proposed mechanisms linking gut microbiota dysbiosis, MGO metabolism, and age-related metabolic dysfunction.

Component	Age-Related Alteration	Consequence on MGO/AGE Metabolism	Biological Outcome	References
Gut microbiota composition	Reduced microbial diversity and dysbiosis	Altered microbial metabolite production	Increased susceptibility to metabolic dysfunction and inflammaging	[6,42]
Intestinal barrier	Increased intestinal permeability (“leaky gut”)	Enhanced translocation of microbial products (e.g., LPS)	Chronic low-grade systemic inflammation	[40,48]
Propionate	Altered production during dysbiosis	Modulation of glucose metabolism and insulin sensitivity	Context-dependent effects on metabolic homeostasis	[57,61]
Imidazole propionate (ImP)	Increased production by dysbiotic microbiota	Impairment of insulin signalling pathways	Promotion of insulin resistance	[72]
Hyperglycaemia and insulin resistance	Increased intracellular glucose utilization	Accumulation of triose phosphates (GAP and DHAP)	Enhanced endogenous MGO formation	[62,63]
MGO accumulation	Increased dicarbonyl stress and reduced scavenging	Formation of MGO-derived AGEs	Oxidative stress, inflammation and metabolic dysfunction	[7,65]
Glyoxalase system (GLO1)	Age-related decline in clearance capacity	Reduced MGO clearance	AGE accumulation and pancreatic inflammation	[66]
Bacterial MGO production	Presence of methylglyoxal synthase (MGS) in intestinal bacteria	Local MGO generation from DHAP	Potential contribution to luminal carbonyl stress	[38,67]
Faecal MGO and AGEs	Increased during dysbiosis	Glycation of proteins, lipids and DNA	Barrier dysfunction and inflammatory activation	[68,69]
Gut–metabolism–inflammation axis	Reciprocal amplification between dysbiosis, inflammation and metabolic stress	Sustained MGO production and AGE accumulation	Increased risk of T2DM and age-related metabolic disorders	[7,50]

## Data Availability

No new data were created in this study. Data sharing is not applicable to this article.

## Abbreviations

The following abbreviations are used in this manuscript:

AGEs	Advanced glycation end products
AKRs	Aldo-keto reductases
ALDHs	Aldehyde dehydrogenases
ATP	Adenosine triphosphate
ATF4	Activating transcription factor 4
BAs	Secondary bile acids
BSA	Bovine serum albumin
CEdG	N2-(1-carboxyethyl)-2′-deoxyguanosine
CHOP	C/EBP homologous protein
DAF-16	Abnormal dauer formation protein 16
DCP	Dicarbonyl proteome
DHAP	Dihydroxyacetone phosphate
DJ-1	Protein deglycase DJ-1
EIF2AK2	Eukaryotic translation initiation factor 2 alpha kinase 2
eIF2α	Eukaryotic translation initiation factor 2 alpha
FXR	Farnesoid X receptor
GAP	Glyceraldehyde-3-phosphate
GAPDH	Glyceraldehyde-3-phosphate dehydrogenase
GIP	Glucose-dependent insulinotropic polypeptide
GLP-1	Glucagon-like peptide-1
GLO1	Glyoxalase 1
GLO2	Glyoxalase 2
GM	Gut microbiota
GSH	Reduced glutathione
HDAC1	Histone deacetylase 1
HDAC2	Histone deacetylase 2
HUVECs	Human umbilical vein endothelial cells
IBS	Irritable bowel syndrome
IL-1β	Interleukin-1 beta
IL-17	Interleukin-17
ImP	Imidazole propionate
ISR	Integrated stress response
LPS	Lipopolysaccharide
MASLD	Metabolic dysfunction-associated steatotic liver disease
MG-H1	Nδ-(5-hydro-5-methyl-4-imidazolon-2-yl)-ornithine
MGdG	Methylglyoxal-derived imidazopurinone deoxyguanosine adduct
MGS	Methylglyoxal synthase
MGO	Methylglyoxal
MYOD	Myogenic differentiation 1
MYOG	Myogenin
NADH	Reduced nicotinamide adenine dinucleotide
NADPH	Reduced nicotinamide adenine dinucleotide phosphate
Nav1.8	Voltage-gated sodium channel 1.8
NF-κB	Nuclear factor kappa-light-chain-enhancer of activated B cells
Nrf2	Nuclear factor erythroid 2-related factor 2
PARK7	Parkinsonism associated deglycase 7
PKR	Protein kinase R
RAGE	Receptor for advanced glycation end products
RCS	Reactive carbonyl species
ROS	Reactive oxygen species
RXR	Retinoid X receptor
SASP	Senescence-associated secretory phenotype
SCFAs	Short-chain fatty acids
SGK-1	Serum- and glucocorticoid-induced kinase 1
SOD1	Superoxide dismutase 1
SSAOs	Semicarbazide-sensitive amine oxidases
T2DM	Type 2 diabetes mellitus
TCA	Tricarboxylic acid cycle
TET	Ten-eleven translocation enzyme
TET1	Ten-eleven translocation methylcytosine dioxygenase 1
TET2	Ten-eleven translocation methylcytosine dioxygenase 2
TGR5	Takeda G protein-coupled receptor 5
TMAO	Trimethylamine N-oxide
TNF-α	Tumor necrosis factor alpha
TORC2	Target of rapamycin complex 2
TPI	Triose phosphate isomerase
TUDCA	Tauroursodeoxycholic acid
UDCA	Ursodeoxycholic acid
